# Predictors of Dual Phosphodiesterase Type 5 Inhibitor Therapy in Persons With Erectile Dysfunction and Diabetes

**DOI:** 10.7759/cureus.91566

**Published:** 2025-09-03

**Authors:** Arun Pande, Ashish Jha, KumarPrafull Chandra, Anurag Pathak, Sanjay Kalra

**Affiliations:** 1 Endocrinology, Lucknow Endocrine Diabetes and Thyroid Clinic, Lucknow, IND; 2 Cardiology, Dr Ram Manohar Lohia Institute of Medical Sciences, Lucknow, IND; 3 General Medicine, Chandra Diabetes And Heart Clinic, Lucknow, IND; 4 Community Medicine, Maharaja Suhel Dev Autonomous State Medical College and Hospital, Bahraich, IND; 5 Endocrinology, Bharti Hospital, Karnal, IND

**Keywords:** dual pde5i, erectile dysfunction, obesity, pde5 inhibitors, serum creatinine, tadalafil, type 2 diabetes mellitus

## Abstract

Background

While on-demand phosphodiesterase type 5 inhibitors (PDE5i) remain standard therapy for erectile dysfunction (ED), data are lacking for non-responders, particularly those with diabetes, who require dual phosphodiesterase type 5 inhibitor therapy (DPT), i.e, daily low-dose tadalafil and on-demand PDE5i. This represents one of the first studies addressing this critical gap in evidence.

Aim

To identify the predictors of requirement for dual PDE5i therapy in persons with type 2 diabetes and ED.

Methods

A retrospective study conducted at a diabetes center in Lucknow, India (November 2020-October 2022), involving a total of 5,243 visits by persons with type 2 diabetes, of which 517 were ED-related visits (n=276). After exclusions, 196 on-demand PDE5i users were included, and 72 non-responders received DPT. Predictors were assessed via multivariate logistic regression. Obesity was defined as body mass index (BMI) ≥25 kg/m² (as per Indian guidelines).

Outcomes

The primary outcome was the need for DPT, defined by on-demand failure.

Results

Individuals requiring DPT were older (53 vs. 49 years, p=0.012), had higher creatinine (0.94 vs. 0.9 mg/dL, p=0.033), and had longer diabetes duration (11 vs. 8 years, p=0.005). A significantly higher proportion of individuals with elevated hemoglobin A1c (HbA1c) (p=0.016), increased creatinine levels (p=0.007), and the presence of obesity (p=0.0047) required substantial DPT. Optimal cutoffs were age ≥50, HbA1c ≥8.1%, and creatinine ≥0.82 mg/dL.

Conclusion

Markers such as age, diabetes duration, HbA1c, obesity, creatinine (ADHOC) predict the need for DPT in type 2 diabetes, enabling personalized ED management. Early recognition of these predictors can help clinicians identify persons likely to require DPT, thus avoiding delays in effective treatment. These findings, though requiring validation in larger and more diverse cohorts, provide an important step toward precision medicine in diabetic men with ED.

## Introduction

Erectile dysfunction (ED) affects a significant proportion of persons with diabetes, ranging from one-third to three-quarters [1,2]. This condition often leads to substantial psychological distress [3]. Notably, the guidelines advise against the routine use of daily tadalafil and are in favor of on-demand therapy. This recommendation is somewhat surprising, given that the guidelines acknowledge that the International Index of Erectile Function (IIEF-EF) scores [4] tend to improve with daily tadalafil compared to on-demand treatment (PRN). The same guidelines also recognize the need for personalized treatment, considering that different persons may have varying preferences [5]. However, the conventional approach has been to prescribe PRN over once-a-day (OAD) dosing. 

Unfortunately, this practice can delay the proper management of ED and exacerbate the psychological burden. Obesity contributes to vascular and metabolic dysfunction, potentially exacerbating ED severity [6]. The global fertility decline, exemplified by India's below-replacement total fertility rate (TFR) of 1.9, coincides with a growing diabetes epidemic, one that silently fuels ED in affected men [6]. As noted by us earlier, the dual burden of diabetes and ED, affecting 50-75% of diabetic men, demands innovative therapeutic approaches, including dual phosphodiesterase type 5 inhibitor (PDE5i) therapy (low-dose tadalafil and on-demand PDE5i regimens), to address non-response to treatment and metabolic-fertility linkages [8]. Despite the effectiveness of PDE5is, stigma and cost barriers leave ED undertreated, particularly in low-resource settings, exacerbating reproductive health disparities. ED is both a biomarker for cardiovascular risk and a contributor to infertility, yet research and policy continue to neglect the health needs of men. We conducted this study in response to these concerns. 

This study aims to identify clinical and biochemical predictors that can help clinicians determine, at baseline, which individuals with type 2 diabetes and ED may benefit from dual PDE5i therapy instead of standard on-demand therapy.

## Materials and methods

Study design and setting

This retrospective cohort study was conducted at a single diabetes care center in Lucknow, India. It focused on a specific population: men with type 2 diabetes and ED who had failed on-demand PDE5i therapy.

**Figure 1:**
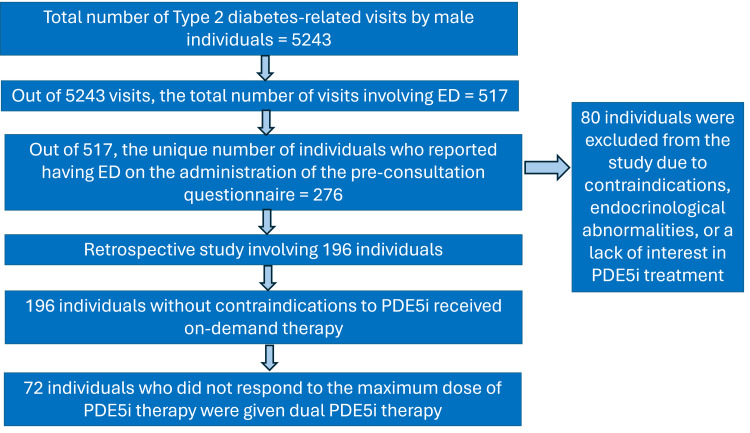
Persons undergoing evaluation PDE5i: Phosphodiesterase-5 inhibitor

The study reviewed data from November 2020 to October 2022, and the data were accessed on 10th February 2025. During this period, there were 5,243 visits by males with type 2 diabetes, of which 517 visits were related to ED. These visits involved 276 unique individuals who reported ED on pre-consultation questionnaires (all individuals), which included inquiries about complications associated with diabetes (Figure 1).

Participant selection

Out of the 276 individuals, 80 were excluded based on the available clinical data. Exclusion criteria included contraindications for PDE5i, lack of interest in PDE5i treatment, or the presence of hypogonadism and other endocrine conditions causing ED.

The identification of endocrine dysfunction was based on clinical presentation (e.g., low libido, loss of body hair, small testicular size, gynecomastia). For individuals exhibiting these signs, targeted hormonal profiling (including, but not limited to, total testosterone, prolactin, luteinizing hormone (LH), and follicle stimulating hormone (FSH)) was performed to confirm a diagnosis. This hormonal workup was conducted based on clinical indication. The final analysis included 196 individuals who had no contraindications and received on-demand or PRN PDE5i therapy. On-demand PDE5i therapy was initiated with one of the following: sildenafil (50-100 mg), tadalafil (10-20 mg), or avanafil (100-200 mg), taken approximately one hour (or 15-30 minutes for avanafil) before anticipated sexual activity. Individuals were titrated to an optimal effective dose, prioritizing the lowest well-tolerated dose that yielded a clinical response. A minimum of four to six attempts at this stabilized dose were required over several weeks to assess treatment efficacy.

Compliance and an adequate trial were verified during a structured follow-up clinic visit with a study physician. This in-person consultation used a standardized checklist to confirm: (1) the number of dosing attempts made, (2) the specific dose and drug taken each time, (3) that the medication was taken on an empty stomach for sildenafil and avanafil users, and (4) that adequate sexual stimulation was attempted after each dose. Only individuals who confirmed ≥4 attempts at the maximum tolerated dose without a satisfactory erection (defined as sufficient for penetration) were classified as non-responders.

Among these, 72 individuals who were confirmed non-responders to the maximum tolerated dose of PRN PDE5i therapy were then prescribed dual PDE5i therapy (DPT). This consisted of a daily low-dose (5 mg) tadalafil regimen in addition to continuing their PRN therapy as needed.

Data source and extraction

Data were extracted from the HealthPlix (Bengaluru, India) Electronic Medical Record (EMR) platform for the specific timeframe of November 2020 to October 2022. The extraction was queried in three-month brackets due to the reporting constraints of the EMR system. The initial data pool identified all individuals within this period whose clinical notes or reports contained the terminology "erectile dysfunction." The raw data was exported into an Excel spreadsheet (Microsoft Corp., Redmond, WA, US) for processing. From this initial dataset, which included all visits, a refined cohort was created by: (1) excluding individuals with any documented cause of diabetes other than type 2, and (2) identifying and consolidating multiple visits to create a dataset of unique persons.

The final analysis was conducted on individual person-level data extracted from this curated dataset, focusing on the 196 individuals who self-reported ED via a predesigned pre-consultation questionnaire administered to all individuals with type 2 diabetes, which included specific queries about ED and other complications. The EMR extraction focused on structured fields capturing demographic details, diabetes-specific variables (e.g., duration, treatment, HbA1c), anthropometric measurements (e.g., BMI, waist circumference), vital signs, prescription records, and laboratory investigations (e.g., lipid profile, serum creatinine). Obesity was defined by Indian guidelines as >25 kg/m^2 ^[9].

To obtain missing information, participants were contacted and asked to complete an online form (Appendices A and B). The form, available in both Hindi and English, included questions on demographic characteristics, lifestyle habits, comorbidities, and diabetes-related complications. It also included a section to obtain consent for participation in the study and publication of the results.

Development of the prediction model

To identify clinical markers predicting the requirement for DPT, we developed a predictive model incorporating parameters significantly associated with therapy, including age, diabetes duration, HbA1c, obesity, and serum creatinine.

Statistical analysis

Data analysis was systematically presented in tabular form to ensure clear interpretation. The Shapiro-Wilk and Kolmogorov-Smirnov tests assessed normality. Categorical variables were summarized as proportions and frequencies, while continuous variables were expressed as means with standard deviations (SD) or medians with interquartile ranges (IQR), depending on distribution. For normally distributed data, the Student’s unpaired t-test was used to compare groups, while the chi-square test evaluated categorical variables. Statistical significance was set at p<0.05.

In the analysis, missing values for continuous variables were imputed using Predictive Mean Matching (PMM), while categorical variables were imputed with logistic regression models. We chose PMM because it is a semi-parametric method that maintains the original distribution of continuous variables and prevents implausible values outside the observed range. For categorical variables, logistic regression is widely recommended because it considers the probabilistic nature of categorical outcomes and stays consistent with the observed data structure. Together, these approaches reduce bias and improve the validity of the final analysis results. Specifically, we used a method with the ‘mice’ package in R statistical software (R Foundation for Statistical Computing, Vienna, Austria, https://www.R-project.org/). The multivariate logistic regression model was employed to determine the combined effect of significant factors on therapy outcomes, with goodness-of-fit assessed using the Hosmer-Lemeshow test. The final model provided adjusted odds ratios to illustrate the relative influence of each predictor. This structured approach ensured the model's robustness and aided in understanding the interplay of variables predicting the requirement for dual PDE5i therapy.

To identify optimal cutoff values for continuous predictors (e.g., age, HbA1c, serum creatinine), we applied Youden’s Index (J). The Youden Index maximizes the sum of sensitivity and specificity (J = sensitivity + specificity − 1) by evaluating all possible cutoff points on the receiver operating characteristic (ROC) curve. The cutoff corresponding to the highest Youden Index was selected as the optimal threshold for dichotomizing each predictor. ROC curves were generated, and the area under the curve (AUC) was reported to assess discriminatory performance.

Ethical considerations

The study was conducted after obtaining approval from the Sanjivni Lung Centre Ethics Committee, Lucknow. All procedures were carried out following Good Clinical Practice (GCP) guidelines and the principles of the Declaration of Helsinki. Participants' data were anonymized to ensure confidentiality, and all identifiable information was securely protected throughout data collection and analysis.

## Results

This study analyzed 196 individuals with type 2 diabetes, categorized based on their response to PDE5i therapy, DPT (n=72) versus PRN (n=124). Individuals requiring DPT had a significantly higher mean age (53 years, SD=8.08) compared to those on PRN therapy (49 years, SD=7.59; p=0.012). The mean eGFR was similar between the two groups (DPT: 108.7, SD=22.07; PRN: 106.7, SD=25.59; p=0.559) (Table 1).

**Table 1:** Description of demographic characteristics and standard protocols DPT: Dual phosphodiesterase type 5 inhibitor therapy; eGFR: estimated glomerular filtration rate; LDL: Low-density lipoprotein cholesterol; HDL: High-density lipoprotein cholesterol; HTN: Hypertension.

S.N.	Variables		Total (n=196); mean±SD	On-demand (n=124); mean±SD	DPT (n=72); mean±SD	P value
1	Age (years)		51.06±7.89	48.96±7.59	52.96±8.08	0.011
2	eGFR (mL/min/1.73m^2^)		107.44±24.32	106.69±5.59	108.72±22.07	0.559
			Median (Q1, Q3)	Median (Q1, Q3)	Median (Q1, Q3)	Wilcoxon Signed Rank test, P value
3	Diabetes duration (years)		9 (5-15)	8 (4-14)	11 (8-15.25)	0.005
4	Weight (kg)		76.0 (69.18-84.05)	75.8 (69.18-83.87)	76.05 (69.22-84.05)	0.903
5	Height (cm)		168.45 (164.0-172.60)	168.25 (164.3-173)	168.7 (162.87-171.1)	0.442
6	BMI (kg/m^2^)		26.57 (24.78-29.44)	26.72 (24.57-29.85)	27.52 (25.06-29.21)	0.977
7	HbA1C (%)		8.35 (7.35-10.20)	8.1 (7.22-10.22)	8.9 (7.5-10.2)	0.140
8	Fasting blood sugar (mg/dL)		149.0 (114.5-189.25)	149 (112.97-192)	151 (125.25-181)	0.562
9	Postprandial blood sugar (mg/dL)		219.45 (150.85-283.5)	224 (179.75-283.5)	203 (150.85-285.5)	0.1389
10	LDL (mg/dL)		63.65 (46.92-97.87)	64.10 (47.0-94.82)	62.65 (45.0-101.23)	0.907
11	HDL (mg/dL)		40.80 (35.0-47.12)	41.07 (36.22-47.12)	40.15 (33.55-47.25)	0.429
12	Creatinine (mg/dL)		0.9 (0.78-1.09)	0.9 (0.75-1.07)	0.94 (0.80-1.10)	0.033
	Presence of comorbidities		n (%)	n (%) 124	n (%) 72	Chi-Square test p value
13	Obesity	Absent (0)	54 (27.55)	38 (30.65)	16 (22.22)	0.203
		Present (1)	142 (72.45)	86 (69.35)	56 (77.78)	
14	HTN	Absent (0)	119 (60.71)	80 (64.52)	39 (54.17)	0.152
		Present (1)	77 (39.29)	44 (35.48)	33 (45.83)	
15	Dyslipidemia	Absent (0)	75 (38.27)	52 (41.94)	23 (31.94)	0.165
		Present (1)	121 (61.73)	72 (58.06)	49 (68.06)	
16	Neuropathy	Absent (0)	89 (45.41)	56 (45.16)	33 (45.83)	0.927
		Present (1)	107 (54.59)	68 (54.84)	39 (54.17)	
17	Retinopathy	Absent (0)	176 (89.8)	113 (91.13)	63 (87.5)	0.418
		Present (1)	20 (10.2)	11 (8.87)	9 (12.5)	
18	Tobacco	Absent (0)	151 (77.04)	93 (75)	58 (80.56)	0.372
		Present (1)	45 (22.96)	31 (25)	14 (19.44)	
19	Smoking	Absent (0)	151 (77.04)	92 (74.19)	59 (81.24)	0.213
		Present (1)	45 (22.96)	32 (25.81)	13 (18.06)	
20	Either smoking or tobacco	Absent (0)	151 (77.04)	92 (74.19)	59 (81.24)	0.213
		Present (1)	45 (22.96)	32 (25.81)	13 (18.06)	
21	Both smoking & tobacco	Absent (0)	152 (77.55)	93 (75)	59 (81.24)	0.261
		Present (1)	44 (22.45)	31 (25)	13 (18.06)	
22	Past tobacco chewer	Absent (0)	149 (76.02)	92 (74.19)	57 (79.17)	0.432
		Present (1)	47 (23.98)	32 (25.81)	15 (20.83)	
23	Past smoker	Absent (0)	149 (76.02)	93 (75)	56 (77.78)	0.660
		Present (1)	47 (23.98)	31 (25)	16 (22.22)	
24	History of either tobacco use or smoking	Absent (0)	149 (76.02)	92 (74.19)	57 (79.17)	0.432
		Present (1)	47 (23.98)	32 (25.81)	15 (20.83)	
25	History of both tobacco chewing and smoking	Absent (0)	149 (76.02)	93 (75)	56 (77.78)	0.660
		Present (1)	47 (23.98)	31 (25)	16 (22.22)	
26	Alcohol consumption	Absent (1)	8 (4.08)	6 (4.84)	2 (2.78)	0.482
		Present (0)	188 (95.92)	118 (95.16)	70 (97.22)	

Significant differences were observed in diabetes duration, with the DPT group having a median of 11 years (IQR = 8-15.3), compared to 8 years (IQR = 4-14) in the PRN group (p=0.005). Similarly, serum creatinine levels were higher in the DPT group (median: 0.94 mg/dL, IQR = 0.8-1.1) versus the PRN group (median: 0.9 mg/dL, IQR = 0.75-1.07; p=0.033). HbA1c levels, although higher in the DPT group (median: 8.9%, IQR = 7.5-10.2) compared to the PRN group (median: 8.1%, IQR = 7.22-10.22), did not reach statistical significance in univariate analysis (p=0.140). However, in multivariate analysis, HbA1c emerged as a significant predictor (p=0.016). Obesity was also a significant predictor in multivariate analysis (p=0.0047) (Appendix C).

Youden index and cutoff values

**Figure 2:**
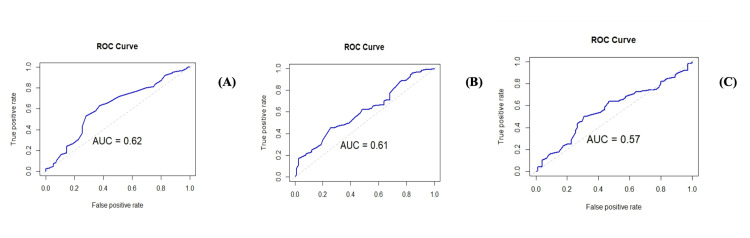
Youden’s index cutoff values for (A) age, (B) creatinine for dual PDE5i therapy, and (C) HbA1C

Using Youden’s index, optimal cutoff values for the significant predictors were identified. Figure 2A-2C show Youden’s Index cutoff value of age, HbA1C, and creatinine, along with Appendix D.

The cutoff values for age (50 years; AUC=0.62), serum creatinine (0.82 mg/dL; AUC=0.61), and HbA1c (8.1%; AUC= 0.57) provide thresholds for distinguishing individuals likely to benefit from DPT.

Obesity and its predictive role

The prevalence of obesity was higher in the DPT group (77.78%) compared to the PRN group (69.35%), though this difference was not statistically significant (p=0.203). Median weight and BMI were also similar between the groups, with no significant differences observed (p>0.9).

Obesity, a key focus of this study, significantly amplified the likelihood of requiring DPT. We used Indian guidelines, which define obesity as BMI ≥25 kg/m² [8].Multivariate analysis revealed that individuals with obesity were five times more likely to require DPT compared to their non-obese counterparts (OR: 5.04; p=0.0047) (Table 2).

**Table 2:** Univariate and multivariate analysis table

Variable	Univariate logistic regression analysis				Multivariate logistic regression analysis				Youden’s index (cutoff values)
	Unadjusted odds ratio (OR)	95% Confidence Interval		P value	Adjusted odds ratio (OR)	95% Confidence Interval		P value	
		Lower limit	Upper limit			Lower limit	Upper limit		
Age	1.05	1.01	1.09	0.011	1.05	0.99	1.10	0.085	50
Diabetes duration (in years)	1.07	1.02	1.12	0.009	-	-	-	-	
HbA1C	1.08	0.94	1.25	0.269	1.32	1.06	1.66	0.016	8.1
Creatinine	4.30	1.42	14.46	0.012	7.72	1.88	37.93	0.007	0.82
Obesity	1.55	0.80	3.10	0.204	5.04	1.70	16.32	0.0047	

Predictive model and performance

**Figure 3:**
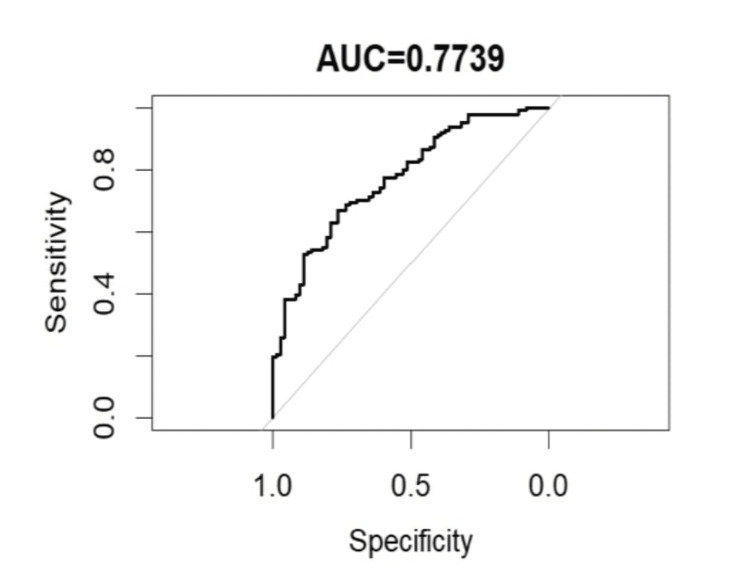
Predictive model for dual PDE5i therapy requirement in individual with diabetes

A predictive model incorporating significant markers, such as age, diabetes duration, HbA1c, obesity, and serum creatinine, was developed to estimate the probability of requiring DPT. While age did not reach statistical significance in the multivariate analysis (p=0.085), it was included in the model due to its clinical relevance. The model demonstrated good discriminative ability, with a C-statistic (AUC) of 0.7739, indicating robust performance (Figure 3).

Probability estimation

**Figure 4:**
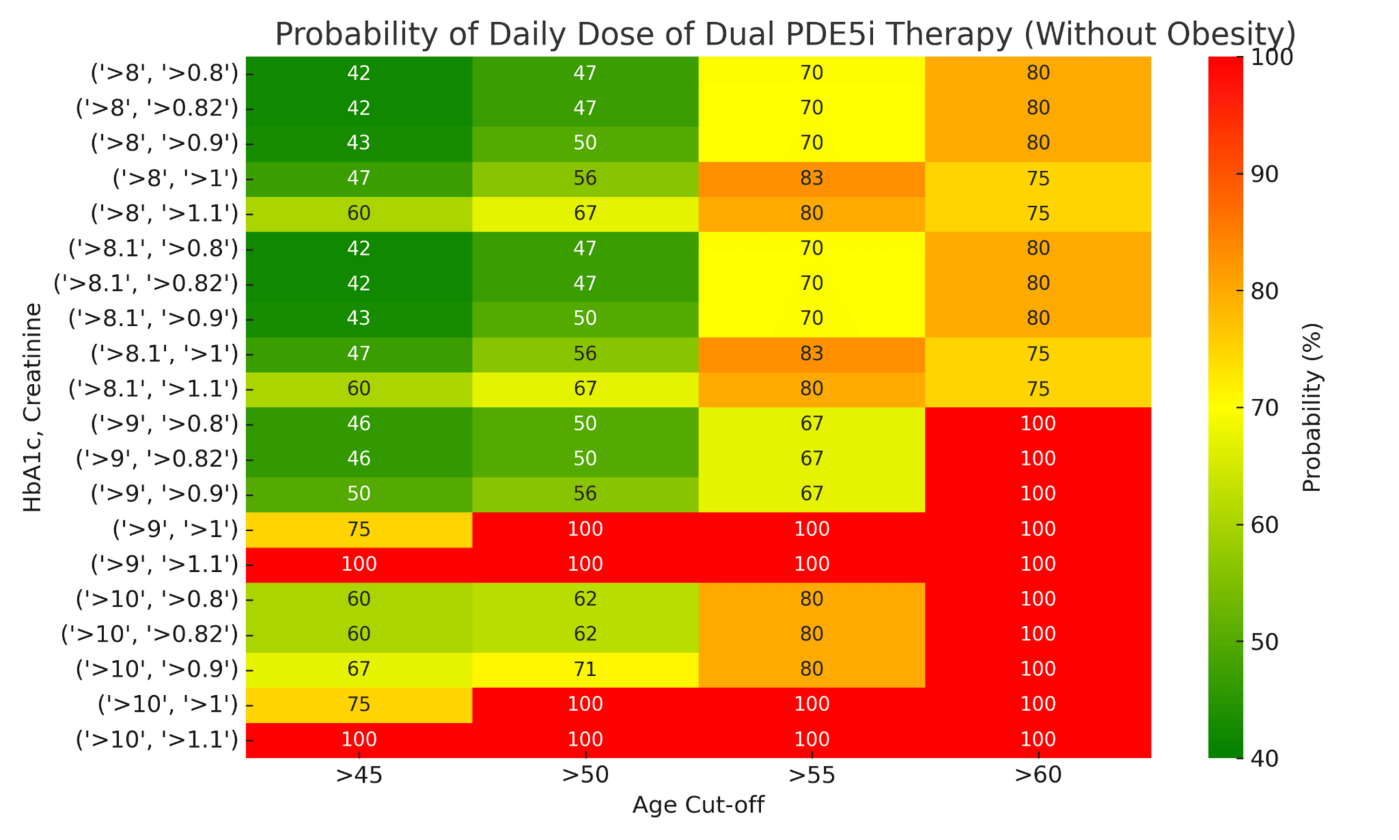
Probability (%) of requiring a daily dose of dual phosphodiesterase type 5 inhibitor therapy (DPT) in individuals without obesity Values stratified by HbA1c (>8%), creatinine levels (>0.8-1 mg/dL), and age cutoffs (45, 50, 55, and 60 years)

**Figure 5:**
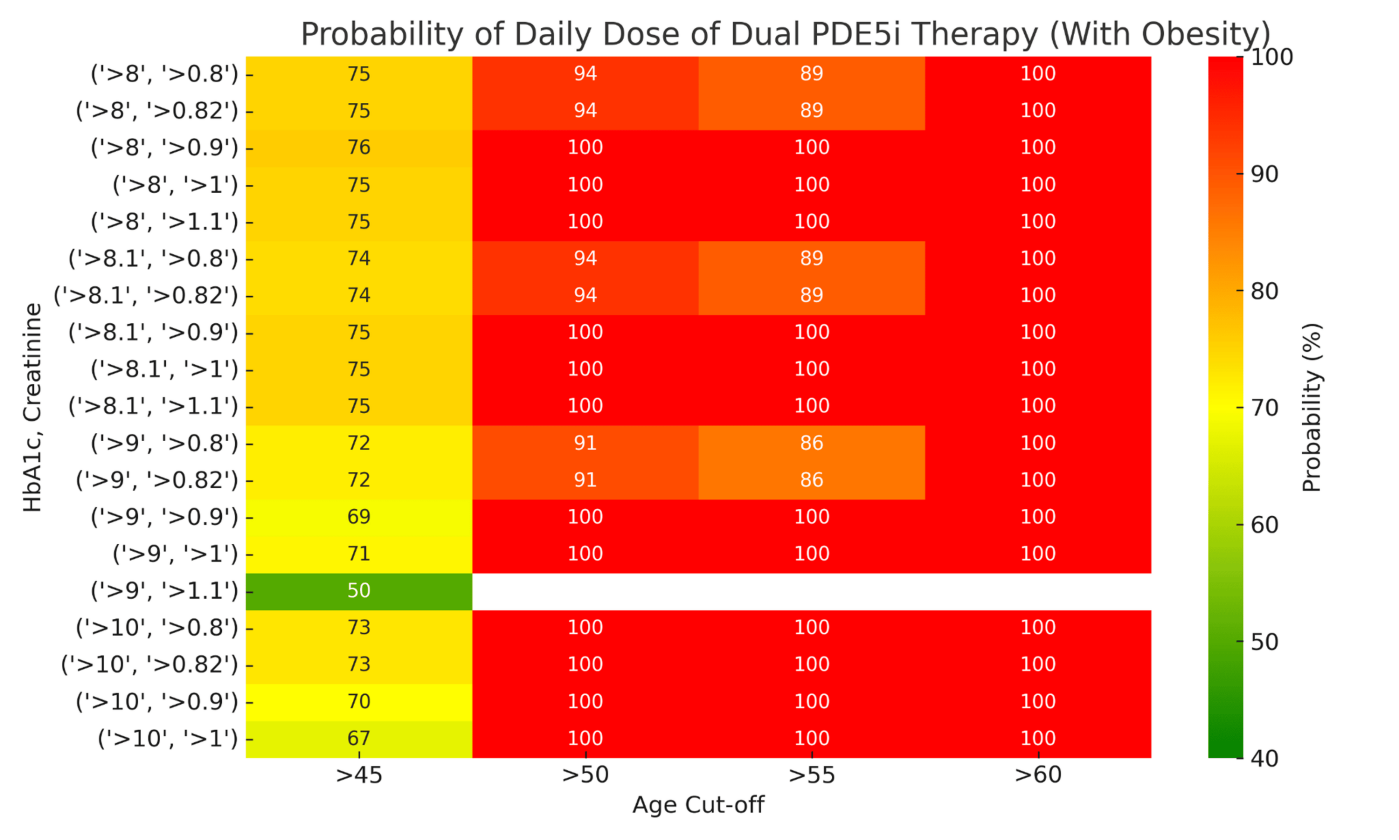
Probability (%) of requiring a daily dose of dual phosphodiesterase type 5 inhibitor therapy (DPT) in individuals with obesity Values stratified by HbA1c (>8%), creatinine levels (>0.8-1 mg/dL), and age cutoffs (45, 50, 55, and 60 years).

Probability estimates based on significant markers demonstrated clear trends. For persons with HbA1c levels >8% and serum creatinine >0.8 mg/dL, the probability of requiring DPT increased with age, reaching 75-100% in those aged ≥60 years. In contrast, for those aged 45-50 with similar biophysical markers, the probability was lower, ranging from 42% to 50%. People with obesity aged ≥55 years with these markers had a 100% probability of requiring DPT, compared to 80-83% in their non-obese counterparts (Figures 4, 5).

These findings underscore the combined influence of age, duration of diabetes, glycemic control, presence of obesity, and renal function on the management of ED using PDE5is. The results highlight the need for a tailored approach, especially for older, people with obesity with suboptimal glycemic and renal profiles.

## Discussion

Our study aimed to distinguish between individuals with diabetes requiring PRN and DPT for ED. It identified age, duration of diabetes, HbA1c, presence of obesity, and serum creatinine (ADHOC parameters) as significant predictors for the necessity of DPT. 

Both our study and the study by Albarakati et al. are retrospective analyses examining factors influencing the effectiveness of PDE5i therapy in individuals with ED. However, there are key differences in the study design and population evaluated. Our study specifically compared the requirement for DPT versus PRN among individuals with type 2 diabetes. In contrast, Albarakati et al. compared responders and non-responders to PDE5i therapy, including both individuals with and without diabetes [10].

The exclusive focus on individuals with type 2 diabetes in our study enabled a more targeted analysis of diabetes-specific factors, among which the ADHOC parameters emerged as significant predictors for DPT. In contrast, Albarakati et al. included a broader population comprising cases both with and without diabetes. This broader scope may account for their identification of hormonal factors, such as low free testosterone and high sex hormone binding globulin (SHBG), as significant predictors, in addition to elevated HbA1c levels. Notably, our study excluded cases with hormonal abnormalities, further refining the focus on diabetes-related markers [10]. These methodological differences underscore the importance of considering specific individual populations when examining predictors of treatment outcomes, as different underlying conditions and characteristics can influence the factors that affect treatment success.

We are not comparing our study with prospective studies, such as the one by Bolat et al. (2018), where the baseline characteristics of study participants are typically similar, making it challenging to discern which characteristics might favor the use of DPT over PRN therapy. Bolat et al. evaluated low-dose daily versus on-demand high-dose tadalafil in individuals with diabetes and erectile and ejaculatory dysfunction [11]. In contrast, our study seeks to identify specific clinical characteristics that can inform practical decision-making. By understanding these markers, clinicians can better determine when DPT might be a more suitable option than PRN therapy, thereby enhancing personalized care for individuals with ED.

Age, serum creatinine, estimated glomerular filtration rate (eGFR), and the requirement for DPT

In our study, age and serum creatinine emerged as significant predictors of the requirement for DPT, whereas eGFR did not show a similar correlation. This finding brings forth some intriguing considerations. Age, creatinine, and weight are critical determinants of eGFR, which is a widely used measure of renal function [12]. However, despite the significant association of age and creatinine with the need for DPT, the mean eGFR values were similar in both groups (106.7 in the PRN group and 108.7 in the DPT group), indicating that both groups were within chronic kidney disease (CKD) stage 1 [13].

The presence of obesity, which was more common in individuals requiring DPT, offers a partial explanation. Obesity is associated with higher eGFR values, which could result in both groups showing similar eGFR despite there being differences in renal health and vascular risk profiles. Obesity contributes to increased renal blood flow and glomerular hyperfiltration, which might mask underlying renal dysfunction when only eGFR is considered [14]. Furthermore, obesity is linked to increased pathophysiological risk factors for vascular diseases, potentially exacerbating conditions like ED [15]. This may explain why individuals with similar eGFR values but differing obesity status exhibit different needs for PDE5i therapy.

However, the association between higher serum creatinine levels and the requirement for DPT cannot be entirely attributed to obesity and eGFR alone. While serum creatinine levels were within the normal range for both groups, they tended to be in the upper half of the normal range in the DPT group compared to the PRN group. We believe this association of serum creatinine is not a chance finding, as our previous study to find the prevalence of ED in individuals with diabetes found a significant association of creatinine with it [2].

We are aware that the urine microalbumin-creatinine ratio, although within normal limits, shows a higher progression to CKD in individuals whose ratios are in the upper half of the normal range [16]. Similarly, individuals with serum creatinine in the upper half of the normal range may have more underlying pathophysiological risk factors for ED than those with values in the lower half. These individuals might experience subclinical renal or vascular changes that are not reflected in eGFR but could predispose them to ED, thereby necessitating DPT.

This hypothesis suggests that serum creatinine, even within normal limits, could serve as an early indicator of potential renal or vascular issues. Recognizing the implications of higher serum creatinine levels could lead to more tailored approaches in the management of ED, emphasizing the importance of evaluating both traditional renal function markers and more subtle indicators of renal and vascular health. Future research should further explore these associations to better understand the underlying mechanisms and improve therapeutic strategies for people with ED.

Our study further quantified the probability of DPT requirement based on age, diabetes duration, HbA1c levels, obesity status, and serum creatinine. Notably, older people with elevated HbA1c and serum creatinine had a substantially higher probability of requiring DPT, reaching 100% in certain combinations. Obesity further amplified these probabilities across age groups, highlighting the complex interplay between metabolic health, renal function, and ED. 

In a healthcare system where delayed follow-ups are commonplace, relying exclusively on PRN therapy has the potential to exacerbate patient distress and reduce compliance [3]. To address this challenge, a more proactive strategy involving early follow-ups for individuals who may not respond adequately to PRN could be a valuable approach. Integrating these considerations into the healthcare framework may enhance an individual's outcomes and foster a more comprehensive and person-centric approach to medical care.

DPT may offer distinct advantages for people with higher risk parameters of older age, prolonged diabetes duration, poor glycemic control, obesity, and elevated creatinine. Regular monitoring of these ADHOC markers can help identify people who may benefit from early escalation to combined daily and on-demand PDE5i regimens, optimizing ED management. A comprehensive evaluation of renal, metabolic, and vascular health is critical to tailoring therapy, ensuring a person-centric approach that addresses multifactorial contributors to ED.

Beyond an individual's care, the ADHOC framework holds broader public health relevance. By integrating these parameters into community health programs particularly in regions with high diabetes prevalence like India, clinicians and policymakers can promote early intervention for diabetes-related complications (neuropathy, retinopathy, and cardiovascular disease). This proactive strategy may reduce the systemic burden of diabetes while improving quality of life through timely ED management. Future research should validate the ADHOC criteria in multicenter diverse populations to refine risk stratification and therapeutic guidelines. 

Strengths and limitations

A key strength of this study lies in addressing the existing divide in literature which favors either PRN or DPT therapy by uniquely focusing on identifying clinical markers (ADHOC parameters) that predict the need for DPT. Unlike prior studies that directly compare PRN vs. DPT dosing at the outset, our work is among the first to characterize individuals with type 2 diabetes and ED based on their response to PDE5i therapy. Importantly, the ADHOC markers not only guide ED management but may also hold broader relevance in assessing microvascular complications, such as autonomic neuropathy, sensory neuropathy, and retinopathy risk stratification. For instance, elevated HbA1c and serum creatinine are established indicators of microvascular damage [16,17] while obesity and prolonged diabetes duration correlate with neuropathy and retinopathy progression [18,19]. This dual applicability underscores the clinical versatility of the ADHOC parameters, offering a framework for holistic diabetes care that addresses both ED and systemic complications. By integrating these markers into routine practice, clinicians may adopt a proactive, person-centered approach to mitigate multifactorial risks in diabetes management.

This study is not without limitations. First, its retrospective, single-center design conducted in an urban Indian setting with a small sample size of non-responders limits the generalizability of our findings to other populations with different genetic and environmental backgrounds. This design may also introduce selection and recall bias, especially concerning the retrospective self-reported data on ED and comorbidities collected via online forms. Second, we had limited control over potential confounding variables, such as psychological factors (e.g., depression, anxiety), detailed concurrent medications, and comprehensive hormonal profiles beyond the excluded conditions; these unmeasured factors could influence ED treatment response. Third, ED was assessed through person-reported satisfaction rather than validated tools, such as the IIEF [4]; future prospective studies incorporating standardized instruments are warranted. Finally, the proposed ADHOC thresholds, including the age cutoff of 50 years, require validation in diverse populations with varying diabetes epidemiology. Despite these limitations, the findings highlight a novel approach to identifying individuals who may benefit from dual PDE5i therapy, offering a foundation for prospective investigations into personalized ED management in diabetes.

## Conclusions

Our study identifies that ADHOC parameters could help identify the requirement for DPT in individuals with type 2 diabetes and ED. These findings underscore the importance of tailoring ED management to individual profiles. Notably, the association between elevated serum creatinine (even within normal ranges) and the requirement for DPT highlights the potential role of subclinical renal impairment in influencing ED severity and treatment strategies.

By translating these findings into clinical practice and public health strategies, healthcare providers may improve treatment efficacy, patient outcomes, and population-level diabetes care.

## Appendices

Appendix A

Online Form Sent to the Individual to Collect the Missing Data (in English)

1. What is your LE number? 

2. What is your full name? 

3. What is your age? 

4. What is your height? 

5. What is your weight? (in kilograms)? 

6. Do you have high blood pressure (hypertension)? 

● Yes 

● No 

7. Are you currently taking medication for high blood pressure? 

● Yes 

● No 

8. Do you have retinopathy (a disease related to diabetes)? 

● Yes 

● No 

9. Do you have heart disease? 

● Yes 

● No 

10. Have you ever had a heart attack?

● Yes 

● No 

11. Have you ever had a stroke? 

● Yes 

● No 

12. Do you smoke currently? 

● Yes 

● No 

13. Have you smoked in the past? 

● Yes 

● No 

14. Do you use any form of tobacco? 

● Yes 

● No 

15. Have you used any form of tobacco in the past? 

● Yes 

● No

Appendix B

Online Form Sent to the Individual to Collect the Missing Data (in Hindi)

 1. आपका LE नंबर या है? 

 2. आपका पूरा नाम क्या है?

3. आपकी उम्र क्या है? 

 4. आपका कद (ऊंचाई) क्या  है? 

 5. आपका वजन कितना है? (किलो ​​मे) ? 

 6. क्या आपको उच रतचाप (हाइपरटशन) है? 

● हां 

● नहीं  

 7. क्या आप वर्त्तमान मे उच्च रक्तचाप की दवा ले रहे हैं ?

● हां 

● नहीं 

8. क्या आपको रेटिनोपथी ( डायबिटज मे होने वाली बीमारी) है?  

● हां 

● नहीं  

9. क्या आपको ह्रदय  रोग है? 

● हां 

● नहीं 

10. क्या आपको कभी दिल का दौरा पड़ा है? 

● हां 

● नहीं 

11.  आपको कभी लकवा (फालिश)  की बीमारी हुई है?

● हां 

● नहीं 

12. क्या आप धूम्रपान  करते? 

● हां 

● नहीं 

13. क्या  आप पहले धूम्रपान करते थे? 

● हां 

● नहीं 

14. क्या  आप किसी भी तंबाकू का सेवन करते है ?  

● हां 

● नहीं 

15. क्या आप पहले किसी भी तंबाकू का सेवन करते थे? 

● हां 

● नहीं 

Appendix C

**Table 3:** Univariate and multivariate analysis table eGFR: estimated glomerular filtration rate; LDL: Low-density lipoprotein cholesterol; HDL: High-density lipoprotein cholesterol; HTN: Hypertension.

Variable	Univariate logistic regression analysis				Multivariate logistic regression analysis			
	Unadjusted Odds Ratio (OR)	95% Confidence Interval		P value	Adjusted Odds Ratio (OR)	95% Confidence Interval		P value
		Lower limit	Upper limit			Lower limit	Upper limit	
Age (years)	1.05	1.01	1.09	0.011	1.05	0.99	1.10	0.085
Diabetes duration (years)	1.07	1.02	1.12	0.009	-	-	-	-
Weight (kg)	0.99	0.96	1.01	0.409	-	-	-	-
Height (cm)	0.98	0.93	1.02	0.266	-	-	-	-
BMI (kg/m^2^)	0.96	0.89	1.04	0.401	-	-	-	-
HbA1C (%)	1.08	0.94	1.25	0.269	1.32	1.06	1.66	0.016
Fasting blood sugar (mg/dL)	1.00	1.00	1.00	0.969	-	-	-	-
Post prandial blood sugar (mg/dL)	1.00	0.99	1.00	0.228	-	-	-	-
LDL (mg/dL)	1.00	0.99	1.01	0.476	-	-	-	-
HDL (mg/dL)	1	0.98	1.01	0.74	-	-	-	-
Creatinine (mg/dL)	4.3	1.42	14.46	0.012	7.72	1.88	37.93	0.007
eGFR (mL/min/1.73m^2^)	1	0.99	1.02	0.573	-	-	-	-
Obesity	1.55	0.8	3.1	0.204	5.04	1.7	16.32	0.0047
HTN	1.54	0.85	2.79	0.154	-	-	-	-
Dyslipidemia	1.54	0.84	2.86	0.116	-	-	-	-
Neuropathy	0.97	0.54	1.75	0.927	-	-	-	-
Retinopathy	1.47	0.56	3.74	0.42	-	-	-	-
Tobacco	0.72	0.35	1.45	0.373	-	-	-	-
Smoking	0.63	0.3	1.28	0.215	-	-	-	-
Either smoking and tobacco	0.63	0.3	1.28	0.215	-	-	-	-
Both smoking and tobacco	0.66	0.31	1.34	0.263				
Past tobacco chewer	0.76	0.37	1.5	0.432	-	-	-	-
Past smoker	0.86	0.42	0.1.69	0.66	-	-	-	-
History of either tobacco use or smoking	0.76	0.37	1.5	0.432	-	-	-	-
History of both tobacco chewing and smoking	0.86	0.42	1.69	0.66	-	-	-	-
Alcohol consumption	0.56	0.08	2.52	0.487	-	-	-	-

Appendix D 

**Table 4:** Measure of diagnostic accuracy of significant variables PDE5i: phosphodiesterase type 5 inhibitor; LR+: Positive likelihood ratio; LR- : Negative likelihood ratio; PPV: Positive predictive value; NPV: Negative predictive value; AUC: Area under the curve.

S. No.	Variables	HbA1c		Creatinine		Obesity	
1	Cutoff	>8.1	<=8.1	>0.82	<=0.82	Absent	Present
2	Dual PDE5i	48	24	51	21	38	86
3	On demand	62	62	68	56	16	56
Measure of diagnostic accuracy	Sensitivity	44%		43%		64%	
	Specificity	72%		73%		45%	
	PPV	67%		71%		91%	
	NPV	50%		45%		12%	
	Precision	67%		71%		91%	
	Accuracy	56%		55%		62%	
	LR+	1.57		1.59		1.16	
	LR-	0.77		0.78		0.8	
	AUC	0.436		0.408		-	
